# Many pitfalls in diagnosis of acute intermittent porphyria: a case report

**DOI:** 10.1186/s13104-018-3615-z

**Published:** 2018-08-02

**Authors:** N. L. R. Indika, T. Kesavan, H. W. Dilanthi, K. L. S. P. K. M. Jayasena, N. D. P. D. Chandrasiri, I. N. Jayasinghe, U. M. T. Piumika, D. M. Vidanapathirana, K. D. A. V. Gunarathne, M. Dissanayake, E. Jasinge, W. Kodikara Arachchi, D. Doheny, R. J. Desnick

**Affiliations:** 1 Department of Biochemistry, Faculty of Medical Sciences, University of Sri Jayewardenepura, Nugegoda, 10250 Sri Lanka; 2 Department of Chemical Pathology, Lady Ridgeway Hospital for Children, Colombo, Sri Lanka; 3 Teaching Hospital Karapitiya, Galle, Sri Lanka; 4 Department of Genetics and Genomic Sciences, Mount Sinai School of Medicine, New York, USA

**Keywords:** Acute intermittent porphyria, Acute porphyria, Hydroxymethylbilane synthase, Misdiagnosis, Porphobilinogen, Mutation analysis, Genetic counseling

## Abstract

**Background:**

Acute intermittent porphyria is a rare autosomal dominant disorder caused by a deficiency of the enzyme, hydroxymethylbilane synthase. Recognition of acute neurovisceral attacks can be difficult due to the nonspecific nature of symptoms.

**Case presentation:**

We report a case of 33-year-old male patient who presented with recurrent episodes of severe abdominal pain, nausea, vomiting, constipation and numbness of bilateral lower limb extremities. These nonspecific neurovisceral attacks were subject to medical and surgical misdiagnoses of acute appendicitis, sinus tachycardia, renal calculi, drug-induced acute interstitial nephritis and two episodes of partial intestinal obstruction. The sixth acute attack raised the suspicion of an acute porphyria. Watson and Schwartz test was positive for porphobilinogen in urine. Mutation analysis by DNA sequencing of the extracted DNA of the proband revealed a previously reported missense mutation, c.517C>T encoding p.R173W in the *HMBS* gene, confirming the diagnosis of Acute Intermittent Porphyria. Four out of five family members who underwent targeted mutation analyses were mutation-positive.

**Conclusion:**

The most common clinical presentation of Acute Intermittent Porphyria is abdominal pain with neurovisceral manifestations which are common to several medical, psychiatric and surgical pathologies. This leads to underdiagnosis and misdiagnosis of this disorder, incorrect management, and severe complications. Therefore, a high index of suspicion and awareness of front line laboratory investigations are important for diagnosis. Definitive diagnosis enables implementation of strategies to prevent acute attacks, and also triggers genetic testing and genetic counseling of at-risk family members.

## Background

Acute intermittent porphyria (AIP) is an autosomal dominant acute porphyria which is caused by mutations in the gene encoding hydroxymethylbilane synthase (HMBS), the third enzyme in the heme biosynthesis pathway. The name of this enzyme is used synonymously with porphobilinogen deaminase (PBGD) [1]. To date, over 400 mutations in the *HMBS* gene have been identified [2].

The most common clinical symptom is acute severe abdominal pain. In acute attacks patients present with neurovisceral manifestations including vomiting, diarrhea, constipation, muscle weakness, numbness, urinary incontinence or retention, palpitations, tremors and seizures as well as behavioral changes like irritability, insomnia, and emotional lability [1, 3, 4].

## Case presentation

A 33-year-old male patient presented to a tertiary care hospital with intermittent abdominal pain, nausea, vomiting, constipation and numbness of bilateral lower limb extremities, of 3 days duration. He was conservatively managed in a surgical unit as partial intestinal obstruction and was awaiting diagnostic laparoscopy. The patient developed confusion and found to have systemic hypertension, and, therefore, was transferred to a medical ward for further management. He is a non-diabetic and did not have a previous history of hypertension. The drug history revealed usage of over-the-counter analgesics for 6 weeks.

The past medical and surgical history revealed similar neurovisceral attacks requiring five acute hospital admissions over 2 years, which ended up in questionable diagnoses. The sixth acute attack raised the suspicion of an acute porphyria. The first attack in January 2013 led to a diagnosis of appendicitis. Because the symptoms worsened following the surgery an emergency laparoscopic exploration was done. But the exploration revealed no cause to explain the worsening symptoms. The second attack was managed as sinus tachycardia and he was started on beta adrenergic blockers. The third attack which was associated with a fever was conservatively managed for a questionable renal colic. Forth attack was complicated with transient hyponatremia and transiently high serum creatinine levels. These complications were attributed to a questionable interstitial nephritis based on the fact that patient had used 50 mg of diclofenac sodium twice a day for 6 weeks, repeating the prescription given by a general practitioner. Another attack in 2015 was managed as partial intestinal obstruction and diagnostic laparoscopy was done. In all these presentations, findings from the ultra sound scans and diagnostic laparoscopy did not support a diagnosis of intra-abdominal pathology.

On examination he was thin built (BMI = 20 kg/m^2^) and pale. Brachial blood pressure was 160/90 mmHg. There were scars of previous appendectomy and laparoscopy surgeries on the abdominal, but, otherwise, the abdominal examination was unremarkable. Muscle power was 4/5 in all four limbs (could not move against a good resistance).

The laboratory investigations performed during this admission showed severe hyponatraemia of 115 mmol/L (136–145) with serum osmolality of 255 mOsmol/Kg (275–295) and urine osmolality of 460 mOsmol/Kg (50–1200 mOsmol/Kg). Serum creatinine concentration was 106 µmol/L (80–115) with blood urea level of 20 mg/dL (6–20). Hemoglobin concentration was 8.2 g/dL (13.5–17.5) and the red cell morphology was normochromic and normocytic. The total cholesterol level was 282 mg/dL (5th to 95th centile; 142–258) with LDL fraction of 225 mg/dL (5th to 95th centile; 78–185). Serum ferritin level was 646 ng/mL (20–250). Arterial blood gas analysis was suggestive of a metabolic acidosis. Echocardiogram showed evidence of left ventricular hypertrophy. There were no significant radiological findings in abdominal X-ray film or abdominal ultrasonography. Blood lead concentration was 3 μg/dL (< 5 μg/dL).

**Fig. 1:**
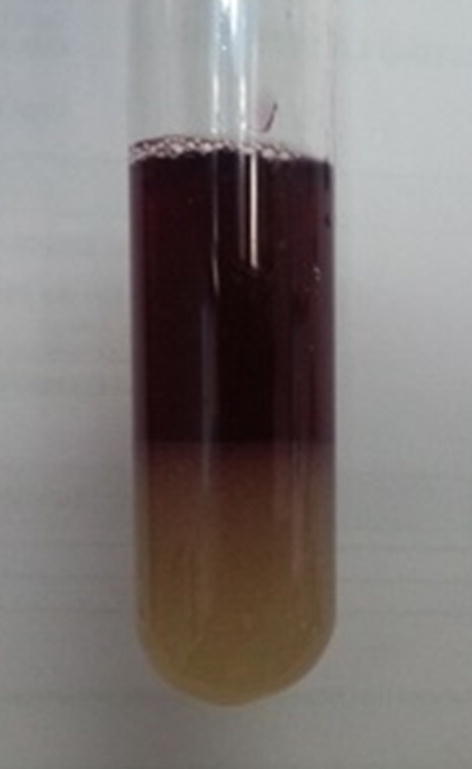
Positive Watson and Schwartz test of the patient; the upper layer is red because porphobilinogen-Ehrlich complex extracts into the upper, aqueous layer

**Fig. 2:**
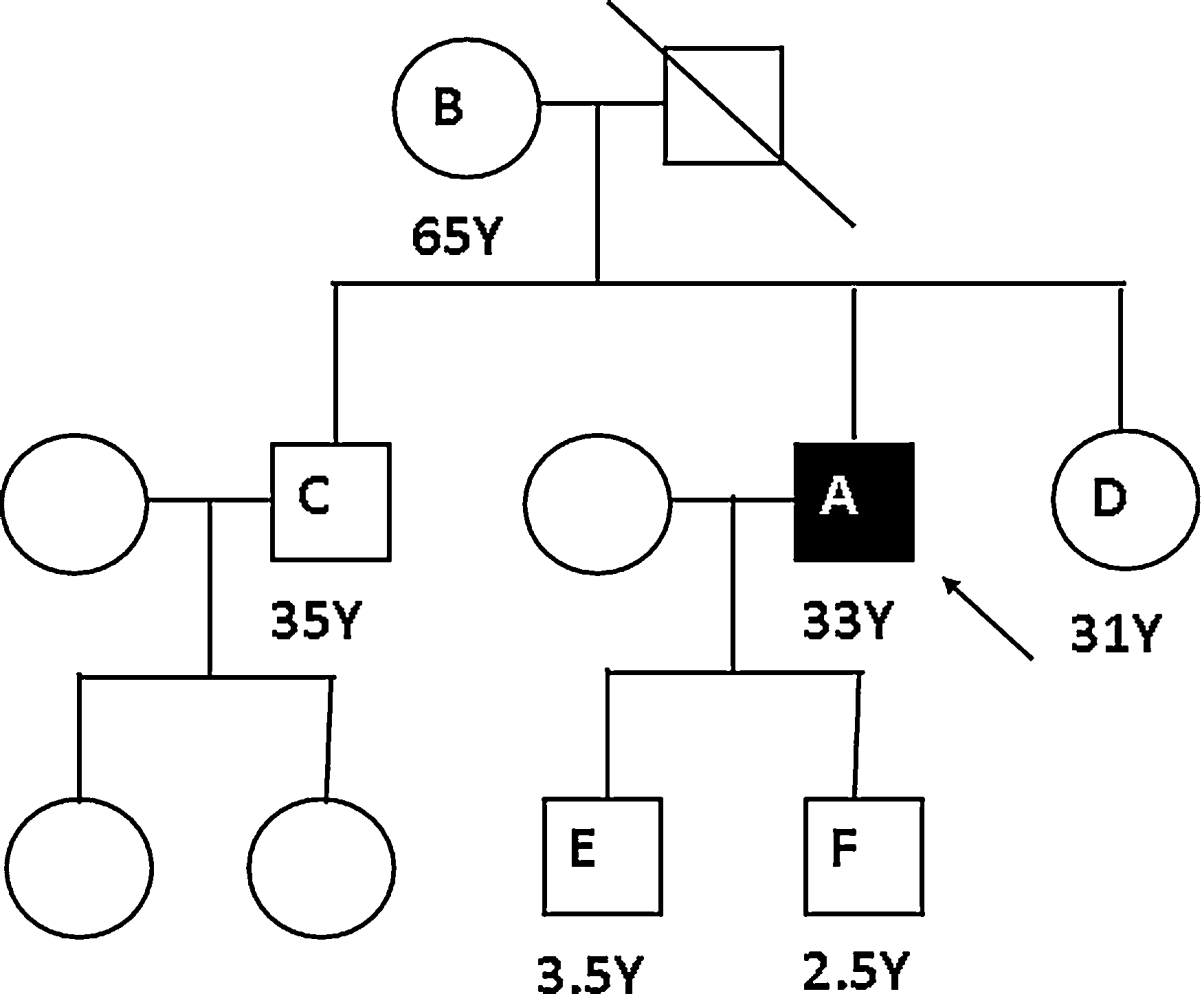
Pedigree of the patient; except the 31-year-old sister, all other first degree relatives of the patient were mutation positive

A urine sample collected during the acute attack was sent to the Department of Chemical Pathology for biochemical analyses. On standing the urine sample gradually turned dark brown. The Watson and Schwartz test for urinary porphobilinogen (PBG) was positive (Fig. 1). Spectrophotometry of urine for total porphyrins showed a “Soret band”. Urine total porphyrin level, calculated using Allen corrected absorbance of the urine sample was 5505.5 nmol/L (< 300 nmol/L). Genetic studies were carried out in an overseas laboratory. The full analysis of *HMBS* gene was performed by PCR amplification of extracted DNA followed by exon specific primer extension analysis of all exons, exon intron boundaries and promotor regions. The gene analysis revealed a previously reported missense mutation, c.517C>T encoding p.R173W in the *HMBS* gene. Targeted mutation analysis was performed by PCR amplification of extracted DNA followed by allele specific primer extension analysis, in five first-degree relatives. Among these, four were heterozygous for the same *HMBS* gene mutation (Fig. 2 and Table 1).

**Table 1:** Clinical and molecular findings of the proband and first-degree relatives

	Family	Age (years)	Clinical status	*HMBS* gene mutation
A	Proband	33	Symptomatic	Present (heterozygous)
B	Mother	65	Hypertension, Impaired renal function	Present (heterozygous)
C	Brother	35	Asymptomatic	Present (heterozygous)
D	Sister	31	Asymptomatic	Absent
E	First son	3.5	Asymptomatic	Present (heterozygous)
F	Second son	2.5	Asymptomatic	Present (heterozygous)

Since heme arginate is not available in Sri Lanka the patient was managed only symptomatically. Carbohydrate loading with intravenous dextrose and oral carbohydrates was the only feasible option. All the medications used for symptomatic management were checked for safety in acute porphyrias. Patient was discharged from the ward after symptoms gradually improved over 6 days to a degree that he can be managed as an out-patient. Response to treatment could not be assessed due to unavailability of quantitative tests to measure urinary aminolevulinic acid (ALA) and PBG in Sri Lanka. The patient was educated regarding precipitating factors of acute porphyria. A diagnostic card with information regarding medications to avoid was provided to the patient. Patient was followed up at the clinic with regular renal functions, hemoglobin and blood pressure monitoring. Follow up of the patient over 1 year following diagnosis revealed that patient suffered from two mild attacks which didn’t require in-patient management. Nerve conduction studies were not carried out because neurological symptoms were not observed in-between acute attacks.

Pre-symptomatic relatives who inherited the *HMBS* mutation were also advised to avoid the trigger factors of acute attacks such as certain medications, fasting, alcohol and hormones. The brother of the proband was counseled regarding the risk of his children inheriting the *HMBS* mutation and recommended targeted mutation analyses for both children.

## Discussion and conclusions

Even though AIP is the most common type of acute hepatic porphyria the estimated prevalence of the disease in Europe countries is 5.4 in per million [5]. There is no data available regarding the prevalence of the disease in Sri Lanka. Review of literature did not disclose any previous Sri Lankan case reports of a genetically confirmed patient with AIP. Since porphyrias are uncommon the clinicians rarely acquire familiarity with these disorders and the curriculum of medical schools focuses very little on these disorders.

Watson and Schwartz test is a screening test for urine PBG. 2 mL of urine is mixed with equal amount of Ehrlichs reagent. If PBG is present it will form porphobilinogen- Ehrlich complex, a red chromogen which is characteristically insoluble in chloroform and N-butanol. When chloroform is added PBG extracts into and gives a red color to the upper aqueous layer, whereas urobilinogen-Ehrlich complex extracts into the chloroform layer. Since patients in acute attacks excrete large amounts PBG false negative results are rare during an acute attack. Spectrophotometry of acidified urine is a semi-quantitative method which can be used as a screening test, to detect urine total porphyrin levels. All porphyrins display an absorption band near 400 nm. In a setting like Sri Lanka, even though sophisticated methods are not widely available these simple reliable laboratory tests are very useful to initiate a diagnosis of a porphyria [6, 7]. To date, quantitative tests for PBG, ALA, uroporphyrin and coproporphyrin levels and genetic studies for porphyria are not available in Sri Lanka. Definitive diagnosis of porphyria should be carried out in an overseas laboratory. Molecular diagnostic studies are useful not only to confirm a biochemically proven AIP but also to identify at risk family members [8].

It is estimated that the penetrance of acute attacks is approximately 1% in heterozygotes with likely *HMBS* pathogenic variants [9]. R173W mutation has been previously reported worldwide, from Sweden, Russia, Spain, United Kingdom, Japan and China. Compared to other common mutations R173W mutation is likely to have a higher clinical penetrance and increased seriousness. This missense mutation is a C-T substitution in nucleotide 517 in exon 10. Conversion of arginine to tryptophan in the mutant protein results in reduced enzyme activity. Even though erythrocyte PBGD activity can be used as an indicator of disease severity the levels can be normal in some patients having acute attacks [10–16].

The most effective treatment in managing acute attacks is intravenous haem arginate, which is available in Europe and many other countries worldwide but not in Sri Lanka. The precipitating factors should be removed. Carbohydrate loading alone may be helpful in mild attacks. While administering the treatment the urinary excretion of ALA and PBG has to be monitored to assess the response. Supportive management includes narcotic analgesics, antiemetics, anxiolytics, correction of dehydration, monitoring fluid balance and psychological support. Strategies in controlling recurrent acute attacks include avoidance of potential trigger factors and prophylactic hemin infusion [17, 18]. However, this patient encounters the disadvantages such as lack of therapeutic and prophylactic heam arginate and lack of monitoring for response to such treatment with quantitative measurement of ALA and PBG due to unavailability and prohibitive cost of such facilities. Therefore long standing resolution of symptoms in this patient is not feasible.

Patients with acute intermittent porphyria usually present with acute attacks with neurovisceral manifestations which are common to several medical, psychiatric and surgical pathologies. Thus, patients with a porphyria, including the acute porphyrias, can potentially encounter the problem of underdiagnosis and misdiagnosis, severely extending the time between onset of symptoms and confirmed diagnosis, resulting in mismanagement and potentially severe debilitating irreversible effects and even death. Medical misdiagnoses can lead to complications in acute porphyria patients due to the possible prescription of porphyrogenic drugs [19]. Surgical misdiagnoses lead to unnecessary surgical interventions like appendectomy and diagnostic laparoscopy. There have been case reports of AIP presenting with psychosis as the only clinical manifestation as well [20]. Under diagnosis of AIP in this category may have complications in patients due to some antipsychotics which could significantly worsen the symptoms of a patient with an acute porphyria. Therefore, a high index of suspicion and awareness of front line laboratory investigations is important for appropriate diagnosis. Definitive diagnosis of a patient with AIP enables implementation of strategies to prevent acute attacks, and also triggers screening and genetic counseling of at-risk family members.

## Abbreviations

AIP: acute intermittent porphyria
ALA: aminolevulinic acid
BMI: body mass index
DNA: deoxyribonucleic acid
HMBS: hydroxymethylbilane synthase
PBG: porphobilinogen
PBGD: porphobilinogen deaminase
PCR: polymerase chain reaction
